# Impact of illicit opioid use on markers of monocyte activation and systemic inflammation in people living with HIV

**DOI:** 10.1371/journal.pone.0265504

**Published:** 2022-05-05

**Authors:** Anastasia Kholodnaia, Kaku So-Armah, Debbie Cheng, Natalia Gnatienko, Gregory Patts, Jeffrey H. Samet, Matthew Freiberg, Dmitry Lioznov

**Affiliations:** 1 Department of Infectious Diseases and Epidemiology, Academician I.P. Pavlov First St. Petersburg State Medical University, Saint-Petersburg, Russian Federation; 2 Department of Medicine, Section of General Internal Medicine, Boston University School of Medicine/Boston Medical Center, Clinical Addiction Research and Education (CARE) Unit, Boston, MA, United States of America; 3 Department of Biostatistics, Boston University School of Public Health, Boston, MA, United States of America; 4 Department of Medicine, Section of General Internal Medicine, Boston Medical Center, Clinical Addiction Research and Education (CARE) Unit, Boston, MA, United States of America; 5 Biostatistics and Epidemiology Data Analytics Center (BEDAC), Boston University School of Public Health, Boston, MA, United States of America; 6 Department of Community Health Sciences, Boston University School of Public Health, Boston, MA, United States of America; 7 Vanderbilt Center for Clinical Cardiovascular Trials Evaluation (V-C3REATE), Vanderbilt University Medical Center, Cardiovascular Division, Nashville, TN, United States of America; 8 Smorodintsev Research Institute of Influenza, St. Petersburg, Russian Federation; University of Cincinnati College of Medicine, UNITED STATES

## Abstract

**Introduction:**

We hypothesize that illicit opioid use increases bacterial translocation from the gut, which intensifies systemic inflammation.

**Objective:**

To investigate the association between opioid use and plasma soluble CD14 [sCD14], interleukin-6 [IL-6] and D-dimer in people living with HIV (PLWH).

**Methods:**

We analyzed data from the Russia ARCH study–an observational cohort of 351 ART-naive PLWH in St. Petersburg, Russia. Plasma levels of sCD14 (primary outcome), IL-6 and D-dimer (secondary outcomes) were evaluated at baseline, 12, and 24 months. Participants were categorized into three groups based on illicit opioid use: current, prior, and never opioid use. Linear mixed effects models were used to evaluate associations.

**Results:**

Compared to never opioid use, sCD14 levels were significantly higher for participants with current opioid use (AMD = 197.8 ng/ml [11.4, 384.2], p = 0.04). IL-6 levels were also higher for participants with current vs. never opioid use (ARM = 2.10 [1.56, 2.83], p <0.001). D-dimer levels were higher for current (ARM = 1.95 [1.43, 2.64], p <0.001) and prior (ARM = 1.57 [1.17, 2.09], p = 0.004) compared to never opioid use.

**Conclusions:**

Among PLWH, current opioid use compared to never use is associated with increased monocyte activation and systemic inflammation.

## Introduction

Persistent immune activation and systemic inflammation in people living with HIV (PLWH) lead not only to the development of AIDS, but are also responsible for the wide spectrum of serious non-AIDS events [1]. Bacterial structural components and metabolites passed into the bloodstream from the gut, as a result of bacterial translocation, are known to induce immune activation [2–4]. HIV-related preconditions of bacterial translocation include: gut epithelial damage via virus replication, massive depletion of mucosal CD4+ T-cells in the course of the disease and specific microbiota abnormalities [5–8], but there also non-HIV factors known to have an impact on bacterial translocation; opioid use being one such factor.

Direct opioid-dependent mechanisms of microbiota damage and gut barrier disruption have been previously described [9–11]. Indirectly, illicit opioid use is a risk factor for chronic viral hepatitis infection. Viral hepatitis is a known driver of bacterial translocation [12–14]. Opioid receptors are expressed in the gastrointestinal tract where endogenous and exogenous opioids modulate gut motility and secretion. Opioid receptors expressed on the gut mucosal immune cells also play a role in intestinal inflammation [15, 16]. Disordered motility and secretion in combination with local inflammation cause gut barrier impairment [17]. Thus, the intestine is a target where HIV and opioids seem to act together. However, data on the effects of illicit opioid use on intestinal permeability, bacterial translocation, and related immune activation among people with HIV are lacking.

This study’s primary objective was to investigate the association between illicit opioid use and plasma soluble CD14 (sCD14), a biomarker of monocyte activation that indicates the presence in blood of lipopolysaccharide (LPS), a component of the gram-negative bacteria cell wall. Therefore, we used sCD14 as a proxy for assessing both immune activation and bacterial translocation. In PLWH, sCD14 correlates with the rate of HIV disease progression and predicts outcomes [18–20]. Additionally, we assessed levels of inflammation (interleukin-6 [IL-6]) and altered coagulation (D-dimer), markers used to understand the link between illicit opioid use and chronic systemic inflammation. We hypothesized that increased opioid exposure is associated with increased biomarkers of monocyte activation, inflammation, and altered coagulation. An exploratory aim assessed whether concomitant liver injury (liver fibrosis) and co-infections (hepatitis C and B) were potential effect modifiers of the relationship between illicit opioid use and monocyte activation, inflammation, and altered coagulation.

## Methods

### Study participants

Russia ARCH (Alcohol Research Collaboration on HIV/AIDS) is an observational cohort study of PLWH who consume alcohol in St. Petersburg, Russia [21]. Participants were recruited from clinical HIV and addiction care sites, non-clinical sites and through snowball recruitment from 2012 to 2015. Participants were followed for 24 months. They completed surveys and provided blood specimens at baseline, 12 months and 24 months. Participants were excluded if they were not fluent in Russian or had cognitive impairment resulting in an inability to provide informed consent. Inclusion criteria for the current analysis were: 1) age 18 to 70 years old; 2) HIV infection; 3) ART-naive at the time of enrollment; 4) available survey and laboratory data. The study was approved by the institutional review boards of Boston University Medical Campus/Boston Medical Center and First Pavlov State Medical University, St. Petersburg, Russia. All participants provided written informed consent.

### Assessments and laboratory analyses

Data were collected via face-to-face interviews administered by trained research assessors and laboratory analyses of blood samples. Data obtained included demographics, medical history, medication use, HIV history and anthropometric data (height, weight). Opioid use and injection drug use were ascertained using a modified Risk Behavior Survey [22, 23]. Alcohol use in past 30 days was ascertained with the Time-Line Follow Back tool (TLFB) [24], alcohol use disorder was obtained from the Mini-International Neuropsychiatric Interview (MINI) instrument [25] and defined according to the ICD 10 and DSM-IV diagnostic criteria. We include non-steroidal anti-inflammatory drug (NSAID) use as confounder because of its action inhibiting immune cells’ production of pro-inflammatory cytokines [26, 27], and diarrhea syndrome or loose bowel moments due to its potential association with increased intestinal permeability [28]. Data on use of NSAIDs were obtained from Russia ARCH interview questionnaire and self-reported diarrhea syndrome or loose bowel moments were obtained from the interview-based HIV Symptom Index (HIV-SI) [29].

Hepatitis B and C were assessed via self-report and liver fibrosis was assessed using the liver fibrosis-4 (FIB-4) score, AST to platelet ratio index (APRI), as well as ultrasound imaging. The FIB-4 score is calculated as the product of age (years) and aspartate aminotransferase (AST, U/L) divided by the product of platelet count (10^9^/L) and the square root of alanine aminotransferase (ALT, U/L). Participants with FIB4 in the indeterminate range for liver fibrosis (1.45–3.25) received Fibroscan imaging. APRI score is calculated as [(AST / upper limit of normal AST) x 100] / platelets (10^9^/L)]. Significant fibrosis was defined as an APRI score ≥ 1.5 [30–32]. CD4+ T-cell count and HIV-1 RNA were measured using standard clinical techniques.

sCD14 was measured using an ELISA (Quantikine sCD14 Immunoassay, R&D Systems Inc; detectable range 40–3200 ng/ml). IL-6 was measured using the MSD Human IL-6 Ultra-sensitive Single-Plex kit (MesoScale Diagnostics, Rockville MD; a working range 0.091–1498 pg/ml). D-dimer was measured using the STAR automated coagulation analyzer (Diagnostica Stago) using an immuno-turbidometric assay (Liatest D-DI; Diagnostica Stago, Parsippany, NJ).

### Dependent variables

The primary outcome of interest was plasma levels of sCD14. Secondary outcomes were IL-6 and D-dimer plasma levels, both natural log transformed due to skewed distributions.

### Main independent variable: Illicit opioid use

Participants were categorized into three groups based on illicit opioid use: 1) current opioid use, defined as past 30-day opioid use; 2) prior opioid use, defined as ever use, but no use in past 30 days; and 3) never opioid use. In exploratory analyses among participants reporting current illicit opioid use, we used quartiles of self-reported frequency of injection drug use in the past 30 days to assess whether there was a relationship between frequency of injections and sCD14, IL-6, or D-dimer.

### Covariates

Covariates assessed included the following: age, gender, body mass index (BMI), HIV viral load (log_10_ transformed) and self-reported time since HIV diagnosis, diarrhea syndrome or loose bowel movements, use of NSAIDs, alcohol use disorder (abuse and dependence), and heavy alcohol use within the past 30 days. Heavy alcohol use was defined using the National Institute on Alcohol Abuse and Alcoholism guidelines for at risk drinking (i.e., for men ≤65 years old more than 14 drinks per week or 5 drinks per occasion; for all women and men > 65 years old—more than 7 drinks per week or 4 drinks per occasion) [33].

### Potential effect modifiers

Variables representing concomitant liver injury (liver fibrosis) and co-infections (hepatitis C and B) were explored as potential effect modifiers. Advanced liver fibrosis was defined as FIB-4 score ≥3.25, APRI score ≥ 1.5 or Fibroscan measurement ≥ 10.5kPa).

### Statistical analysis

Descriptive statistics were used to characterize the demographic and clinical characteristics of the overall analytic sample, and groups were stratified by opioid use. Characteristics were compared across the three opioid use groups using chi-square and Fisher’s tests for categorical variables and ANOVA/Kruskal Wallis for continuous variables, as appropriate. Skewed outcomes (i.e., IL-6, D-dimer) were log-transformed for regression analyses, then back transformed for ease of interpretation. Linear mixed effects models were constructed to estimate the unadjusted and adjusted associations between opioid use and sCD14, IL-6, and D-dimer, using random intercepts and slopes to account for correlation due to incorporating repeated measures from the same participant over time. Adjusted analyses were conducted to assess the influence of potential confounding factors. The primary model included age, BMI, HIV-1 RNA, time since HIV diagnosis, alcohol consumption and alcohol use disorder. Sensitivity analyses included gender, diarrhea syndrome and use of NSAIDs additionally, as potential confounders. For the primary outcome, sCD14, we report adjusted mean differences (AMD) between those who report never using opioids (referent group) and each of the other two exposure groups (i.e., those reporting current and prior opioid use). For the secondary outcomes IL-6 and D-dimer, adjusted ratios of means (ARM) are reported due to the natural log transformations used in the analyses. We used similar models to estimate associations between frequency of injection drug use among those reporting current opioid use and sCD14, IL-6 and, D-dimer. Lastly, in exploratory analyses, we assessed whether liver disease (hepatitis C or B) and liver fibrosis, modify the association between opioid use and sCD14, IL-6, and D-dimer. Interactions were tested separately for each of the three liver conditions adjusting for age, body mass index, HIV viral load, years from first HIV-positive test, and heavy alcohol use. We used an alpha level of 0.05, except in the case of interaction tests, which were conducted at a level of 0.10 due to the exploratory, hypothesis generating nature of the analyses. All analyses were performed using SAS version 9.3 (SAS Institute, Inc, NC, USA).

## Results

### Participant characteristics

The final analytic sample included 351 participants. Illicit opioid use within the past 30 days was reported by 121 and prior opioid use by 186 participants (Table 1).

**Table 1:** Baseline demographic and clinical characteristics of Russian PLWH recruited 2012–2015.

	Overall n = 351	Never opioid use n = 44	Prior opioid use n = 186	Current opioid use n = 121	p-value
**Demographics**					
Age, mean (SD)	33.7 (5.6)	35.2 (9.1)	33.6 (5.0)	33.3 (4.6)	0.15
Gender, male, n (%)	248 (70.7%)	24 (54.5%)	138 (74.2%)	86 (71.1%)	0.04
BMI, mean (SD)	22.9 (3.1)	23.5 (3.2)	23.0 (3.1)	22.5 (3.1)	0.21
**HIV Disease Characteristics**					
Years since HIV diagnosis,	7.1 (4.7)	2.7 (3.5)	7.7 (4.8)	7.8 (4.2)	< .0001
mean (SD)					
HIV viral load (log10),	4.3 (1.1)	4.4 (0.9)	4.2 (1.1)	4.3 (1.2)	0.52
mean (SD)					
CD4 count, mean (SD)	533 (297)	571 (262)	540 (306)	507 (298)	0.49
Antiretroviral therapy	0	0	0	0	
**Alcohol Substance Use Disorder**					< .0001^†^
Abuse NO, dependence NO	102 (29.1%)	24 (55.8%)	58 (31.2%)	20 (16.5%)	
Abuse YES, dependence NO	30 (8.6%)	2 (4.7%)	16 (8.6%)	12 (9.9%)	
Abuse YES, dependence YES	218 (62.3%)	17 (39.5%)	112 (60.2%)	89 (73.6%)	
**Alcohol consumption**					0.0002^†^
Heavy drinking (past 30 days)	250 (71.2%)	36 (81.8%)	119 (64.0%)	95 (78.5%)	
Moderate drinking (past 30 days)	46 (13.1%)	6 (13.6%)	22 (11.8%)	18 (14.9%)	
Abstinent (past 30 days)	55 (15.7%)	2 (4.5%)	45 (24.2%)	8 (6.6%)	
**infections**					
Chronic Hepatitis C	305 (86.9%)	11 (25.0%)	175 (94.1%)	119 (98.3%)	< .0001
Chronic Hepatitis B	131 (37.3%)	4 (9.1%)	62 (33.3%)	65 (53.7%)	< .0001
Diarrhea or loose bowel movements in past 4 weeks	74 (21.1%)	11 (25.0%)	38 (20.4%)	25 (20.7%)	0.79
**Use of NSAIDs**					
Ibuprofen in past 24 hours	2 (0.6%)	0 (0.0%)	0 (0.0%)	2 (1.7%)	0.15
Aspirin in past 24 hours	12 (3.4%)	2 (4.5%)	7 (3.8%)	3 (2.5%)	0.76
**Baseline plasma concentrations of sCD14, IL-6 and D-dimer**					
Soluble CD14 (ng/mL), mean (SD)	2028 (608)	1915 (577)	1930 (597)	2213 (596)	0.0001
Interleukin 6 (pg/mL),					
Median (25^th^, 75^th^)	0.9 (0.5, 1.6)	0.8 (0.4, 1.1)	0.7 (0.5, 1.4)	1.3 (0.8, 2.2)	0.66
D-dimer (ug/mL),					
Median (25^th^, 75^th^)	0.4 (0.3, 0.7)	0.3 (0.2, 0.5)	0.4 (0.2, 0.6)	0.5 (0.3, 0.9)	0.001
Frequency of injections per month, Median (25^th^, 75^th^)		10 (4;15)			
FIB4, N	244				
Median (25^th^, 75^th^)	1.4 (0.9, 2.2)	1.1 (0.8, 1.6)	1.4 (0.9, 2.3)	1.5 (1.0, 2.4)	0.21
APRI, N	244				
Median (25^th^, 75^th^)	0.6 (0.4, 1.1)	0.4 (0.2, 0.6)	0.6 (0.4, 1.2)	0.6 (0.4, 1.2)	0.21
FIBROSCAN RESULT, N	63				
Median (25^th^, 75^th^)	6.6 (5.3, 7.8)	6.5 (4.7, 7.8)	7.2 (5.6, 8.0)	6.2 (5.3, 7.6)	0.59

Abbreviations: SD, standard deviation

^†^hi-square test

Participants’ mean age was 34 years, 71% were male and mean BMI was 22.9 kg/m^2^. Mean duration since HIV diagnosis was 7 years. All participants were ART-naive at the time of the enrollment. There were no significant differences in peripheral CD4+ T-cell count (mean [SD] - 533[297] cells per uL) and log copies of HIV-1 RNA (mean[SD] 4.3 [1.1]) between opioid use groups. Compared to those reporting never opioid use, participants reporting current and prior opioid use had a higher prevalence of hepatitis C and hepatitis B.

Alcohol use disorder prevalence was higher with opioid exposure (69% current use and 84% prior use), but heavy alcohol use within the past 30 days was common in all three groups. Among those reporting current opioid use, 75% reported 10–15 injection drug use episodes in the prior 30 days. Most participants (75%) had no evidence of advanced liver fibrosis/cirrhosis. Of 63 patients in the indeterminate FIB-4 range, median (IQR) Fibroscan measurement was 6.6 (5.3, 7.8) kPa indicating no or mild liver fibrosis [34].

### sCD14 assessment

In unadjusted analyses sCD14 levels were significantly higher in those with current (mean difference [MD] 342 ng/ml [95%CI: 190, 494], p<0.001) and prior opioid use (MD 161 ng/ml [95%CI: 16.5, 305], p = 0.03) compared to those with never opioid use (Table 2).

**Table 2:** Unadjusted and adjusted difference of means and 95% Confidence Intervals (CI) for the association between opioid use category and plasma concentration of sCD14 among a cohort of Russia PLWH (N = 344).

			Unadjusted Difference of Means (95% CI)	p-value	Main Adjusted Difference of Means (95% CI)	p-value	Sensitivity Adjusted Difference of Means (95% CI)	p-value
**Opioid use category**	Current opioid use (within past 30 days)		342.1 (190.3, 493.9)	< .0001	197.8 (11.4, 384.2)	0.039	259.5 (77.0, 441.9)	0.008
	Prior opioid use		160.8 (16.5, 305.2)	0.0292	47.3 (-127.1, 221.8)	0.579	116.7 (-54.4, 287.7)	0.170
	Never opioid use		REF		REF		REF	
Age (years)					7.4 (-1.9, 16.8)	0.112	10.6 (1.5, 19.8)	0.025
BMI					-23.5 (-38.9, -8.1)	0.005	-18.5 (-33.5, -3.5)	0.018
HIV viral load (log10)					71.9 (32.3, 111.6)	0.001	78.7 (39.5, 117.8)	<0.001
Years since first positive HIV test					11.2 (-0.7, 23.2)	0.065	10.0 (-1.6, 21.5)	0.088
Alcohol MINI result		Abuse & dependence			-4.7 (-117.3, 107.9)	0.996	25.9 (-86.4, 138.2)	0.875
		Abuse, no dependence			-0.2 (-187.6, 187.1)		33.8 (-152.6, 220.2)	
		No abuse, no dependence			REF		REF	
Drinking status (TLFB), 30 days		Heavy			135.6 (14.6, 256.5)	0.086	139.1 (19.2, 259.1)	0.075
		Moderate			98.8 (-42.5, 240.1)		96.5 (-42.9, 235.9)	
		Abstinent			REF		REF	
Sex (female vs. male)							260.8 (145.2, 376.4)	<0.001
Diarrhea, past 4 weeks (yes vs. no)							84.5 (-17.9, 186.9)	0.100
Ibuprofen use, past 24 hours (yes vs. no)							224.1 (-334.7, 782.9)	0.413
Aspirin use, past 24 hours (yes vs. no)							84.6 (-149.7, 318.9)	0.460

In adjusted models, compared to never opioids use, current opioid use was associated with higher sCD14 levels (adjusted mean difference [AMD] 198 ng/ml [95%CI: 11.4, 384], p = 0.039) (Table 2), while those with prior opioid use had similar sCD14 levels (AMD 47.3 [95%CI: -127, 222], p = 0.58).

### IL-6 assessment

In unadjusted analyses IL-6 levels were also significantly higher for current (ratio of means (RM) 2.31 [95%CI: 1.76, 3.06, p<0.001) and prior opioid use (RM 1.53 [95%CI: 1.17, 1.99], p = 0.002) compared to never opioid use (Table 3).

**Table 3:** Association between opioid use category and plasma concentrations of IL-6 among a cohort of Russian PLWH (N = 344).

	IL-6		
	Ratio of Means^†^ (95% CI), p-value		
	current opioid use (within past 30 days)	prior opioid use	never opioid use
Unadjusted	2.313	1.525	REF
	(1.76, 3.05)	(1.17, 1.99)	
	p<0.001	p = 0.002	
Main	2.101	1.311	
	(1.56, 2.83),	(0.99, 1.73)	
Adjusted^‡^			
	p<0.001	p = 0.055	
Sensitivity	2.130	1.333	
	(1.58, 2.88)	(1.01, 1.77)	
Adjusted^§^			
	p<0.001	p = 0.046	

^†^Represents the ratio of means after back transformation for natural log scale

^‡^Linear mixed effects model adjusted for age, body mass index, HIV viral load, years from first HIV-positive test, alcohol consumption (including past 30 day), alcohol use disorder

^§^Linear mixed effects model adjusted for age, body mass index, HIV viral load, years from first HIV-positive test, alcohol consumption (including past 30 day), alcohol use disorder + gender, use of NSAIDs and diarrhea

In adjusted models, compared to never opioid use, we observed significantly higher IL-6 levels with current opioid use (adjusted ratio of means [ARM] 2.10 [95%CI: 1.56, 2.83], p<0.001). The association between prior opioid use and IL-6 was somewhat attenuated in the main adjusted model (ARM 1.31[95%CI: 0.99, 1.73], p = 0.055) and no longer statistically significant. The results were similar for both groups in sensitivity analyses that additionally controlled for gender, use of NSAIDs and diarrhea.

### D-dimer assessment

In unadjusted analyses D-dimer levels were significantly higher with current (RM 1.96 [95%CI: 1.53, 2.51], p<0.001) and prior opioid use (RM 1.62 [95%CI: 1.28, 2.05], p<0.001) compared to never opioid use (Table 4).

**Table 4:** Association between opioid use category and plasma concentrations of D-dimer among a cohort of Russian PLWH (N = 346).

	D-dimer		
	Ratio of Means^†^ (95% CI), p-value		
	current opioid use (within past 30 days)	prior opioid use	never opioid use
Unadjusted	1.96	1.62	REF
	(1.53, 2.51)	(1.28, 2.05)	
	p<0.001	p<0.001	
Main	1.95	1.57	
	(1.43, 2.64)	(1.172, 2.09)	
Adjusted^‡^			
	p<0.001	p = 0.004	
Sensitivity	2.06	1.68	
	(1.52, 2.80)	(1.26, 2.25)	
Adjusted^§^			
	p<0.001	p = 0.001	

^†^Represents the ratio of means after back transformation from natural log scale

^‡^Linear mixed effects model adjusted for age, body mass index, HIV viral load, years from first HIV-positive test, alcohol consumption (including past 30 day), alcohol use disorder

^§^Linear mixed effects model adjusted for age, body mass index, HIV viral load, years from first HIV-positive test, alcohol consumption (including past 30 day), alcohol use disorder + gender, use of NSAIDs and diarrhea

In the main adjusted analyses, D-dimer levels were higher for those with current (ARM 1.95 [95%CI: 1.43, 2.64], p<0.001) and prior opioid use (ARM 1.57 [95%CI: 1.17, 2.09], p = 0.004) compared to those with never opioid use (Table 4). D-dimer levels were also higher with current versus prior opioid use (ARM 1.24 [95%CI: 1.05, 1.46], p = 0.01). The results were consistent in the sensitivity analyses.

### Frequency of injections exploratory assessment

We did not detect a significant association between number of injections per month and sCD14, IL-6 or D-dimer plasma concentrations among those with current opioid use (Table 5).

**Table 5:** Association between frequency of opioid use in past 30 days and plasma concentrations of sCD14, IL-6 and D-dimer among a cohort of Russian PLWH (N = 344).

Times injected in past 30 days (quartile)	sCD14	IL-6	D-dimer
	Difference of Means (95% CI)	Ratio of Means (95% CI)	Ratio of Means (95% CI)
1–3 injections /month [Quartile 1]	REF		
4–10 injections /month [Quartile 2]	73.7 (-168.8, 316.1)	1.17 (0.79, 1.74)	1.20 (0.82, 1.77)
11–21 injections /month [Quartile 3]	192.1 (-36.6, 420.7)	0.98 (0.67, 1.42)	0.79 (0.56, 1.13)
22–90 injections /month [Quartile 4]	-126.4 (-353.5, 100.6)	0.951 (0.66, 1.38)	0.99 (0.69, 1.45)
Type 3 p-value	p = 0.060	p = 0.729	p = 0.227

Linear mixed effects model adjusted for age, body mass index, HIV viral load, years from first HIV-positive test, alcohol consumption (including past 30 day), alcohol use disorder.

In adjusted models, patients who reported 22–90 injections per month had no significant differences in sCD14 (AMD -126.4 [95%CI: -353.5, 100.6]), IL-6 (ARM 0.95 [95%CI: 0.66, 1.37]) and D-dimer (ARM 0.99 [95%CI: 0.69, 1.45]) levels compared to those who reported 1–3 injections per month.

### Concomitant liver diseases

We did not detect interaction effects in our sample between opioid use and liver fibrosis (sCD14, p = 0.258; IL-6, p = 0.341; D-dimer, p = 0.158) or concomitant hepatitis C (sCD14, p = 0.94; IL-6, p = 0.585; D-dimer, p = 0.932) and concomitant hepatitis B (sCD14, p = 0.702; IL-6, p = 0.781; D-dimer, p = 0.867) on the concentration of biomarkers studied.

## Discussion

We found that current opioid use, compared to never use, is associated with elevated sCD14, IL-6, and D-dimer—biomarkers of monocyte activation, systemic inflammation, and altered coagulation, respectively, among people living with HIV. Prior opioid use is also associated with altered coagulation.

Current understanding of how opioid use affects bacterial translocation and further monocyte activation is derived predominantly from animal data [9, 35–37] with few studies done in humans and fewer still among PLWH. This basic science work is summarized in an excellent review by Eisenstein et al [38]. We will focus our discussion on data from human studies.

Ancuta et al. reported that in PLWH, heroin use was associated with high plasma levels of lipopolysaccharide [39], a marker of bacterial translocation. However, heroin was not associated with sCD14, even though sCD14 and LPS were correlated. This 2008 study focused on immunocompromised participants (median CD4+ T-cell count 64, range:1–299 cells/uL) and CD4+ T-cell count differed across categories of substance use. In our study, there were no significant differences in immunological status and HIV-1 viral load across categories of reported opioid use. Since CD4+ T-cell count and HIV-1 viral load are associated with sCD14, these differences could explain the discrepant findings between studies. Given WHO recommendations for immediate initiation of antiretroviral therapy after HIV diagnosis (i.e., without waiting for CD4+ T-cell count to decline), our study results may be more generalizable to contemporary opioid using HIV populations than the study by Ancuta et al.

While current opioid use was associated with IL-6, sCD14 or D-dimer, we did not detect an association between injection frequency and these biomarkers. This may be explained by the importance and variability in the quality and quantity of substance being injected; factors not captured in this analysis. Alternatively, this discrepancy may reflect the different referent groups in these two analyses. In the primary analysis, we used participants who never used opioid as a referent group, while in the secondary analyses the referent group are participants who reported opioid use with a frequency of 1–3 injections per month.

Previous studies show that HIV and HCV co-infection positively correlate with bacterial translocation from the gut and systemic inflammation, mainly as a result of liver fibrosis and concomitant portal hypertension [12–14]. Though our cohort had a high prevalence of HCV co-infection, there was low prevalence of advanced liver fibrosis. This may explain why we did not detect a significant interaction between opioid use and liver fibrosis on the biomarkers we evaluated.

Our study has important potential implications. Chronic systemic inflammation (higher sCD14, IL-6 and D-dimer levels) is associated with increased risk of worse HIV disease outcomes and serious non-AIDS events [40–42]. Future research should assess how alterations in opioid exposure and type of opioids involved impact these inflammatory biomarkers and whether the biomarkers mediate the association between opioid exposure and morbidity or mortality.

Our study had limitations. First, because our opioid use variables were based on self-reported data, some misclassification might have occurred (e.g., participants with current opioid use classified as having prior opioid use). However, this misclassification would only attenuate the association between current opioid use and levels of studied biomarkers. It is less likely that participants with never opioid use would report prior or current opioid use. Secondly, opioid use was not further categorized into specific types, though our prior work suggests heroin and methadone in combination with heroin were the predominant opioids use in this population at the time of this study [43]. Thirdly, we did not assess the actual morphine equivalent doses of opioid use, although we did consider self-reported frequency of injections. Fourthly, data about HCV and HBV infections were self-reported, with no laboratory confirmation of current viral replication and antibody reactivity. We did however measure liver fibrosis using blood and imaging-based modalities, since we believe liver fibrosis (not liver-tropic infections) to be the main confounder/moderator in our analyses. Fifthly, our results could have been confounded by alcohol use. Given alcohol dependent mechanisms of increased bacterial translocation [44, 45], we assessed the characteristics of participants’ alcohol use in our sample. We found that although alcohol use disorder prevalence was especially high among participants with opioid exposure (69% in group of “current opioid use” and 84% “prior opioid use” group vs. 44% in “never opioid use” group), heavy alcohol use within the past 30 days was common in all three groups (79% in “current opioid use” group, 64% in “prior opioid use” group and 81% in “never opioid use” group). Accordingly, our adjusted regression models accounted for both alcohol use disorder and alcohol consumption in the past 30 days, suggesting the observed associations were not confounded by alcohol use. Finally, since Russia ARCH is an observational cohort study, causality cannot be determined and we cannot rule out the existence of unmeasured confounding.

## Conclusions

Opioid use is associated with poor prognoses in people with HIV [46]. The non-ART adherence-related mechanisms for this association are still not fully elucidated. We hypothesize that bacterial translocation is increased in PLWH, who use opioids, leading to activation of immune cells through mononuclear phagocyte system, and chronically increased systemic inflammation, which ultimately leads to worse morbidity and mortality. Our results support this hypothesis and future work should assess the prognostic significance of sCD14, IL-6, and D-dimer with morbidity and mortality among PLWH who use opioids.

In addition, these data support the importance of opioid use disorder treatment for PLWH to preserve the gains in healthy life expectancy from effective HIV treatment. Future studies among PLWH should examine whether opioid exposure is associated with higher rates of non-AIDS events via mechanisms mediated by these immune processes. Other areas for future research include assessing the impact of highly effective medications for opioid use disorder (e.g., methadone, buprenorphine) on gut barrier function, monocyte activation, and inflammation.
